# Supplementary material for: Improving the representativeness of UK’s national COVID-19 Infection Survey through spatio-temporal regression and post-stratification
Source: Nat Commun. 2024 Jun 24;15:5340. doi: 10.1038/s41467-024-49201-4 (PMC11196632; doi:10.1038/s41467-024-49201-4)
Supplement: Supplementary file 1 — Supplementary Information [file 41467_2024_49201_MOESM1_ESM.pdf]

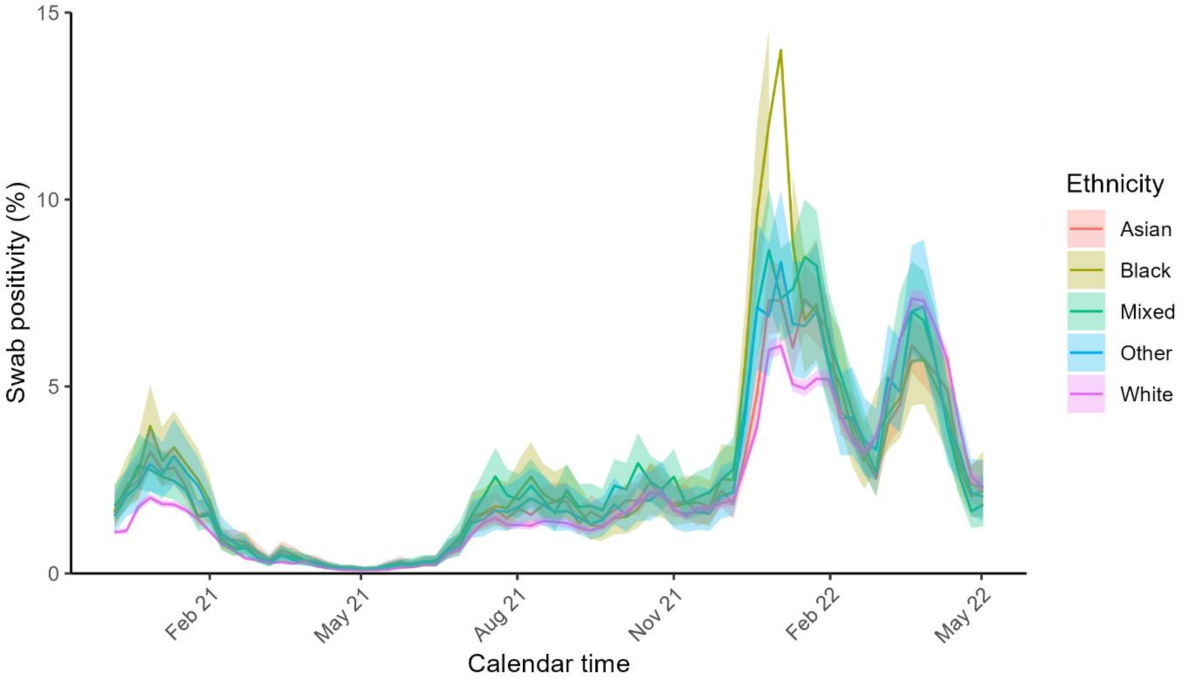

Fig. S1. Post-stratified estimate of swab positivity by ethnicity over time. Estimates are post-stratified for age, sex, CIS area, ethnicity, and vaccination status. Estimates are presented as posterior medians (solid lines) with shading representing 95% credible intervals.

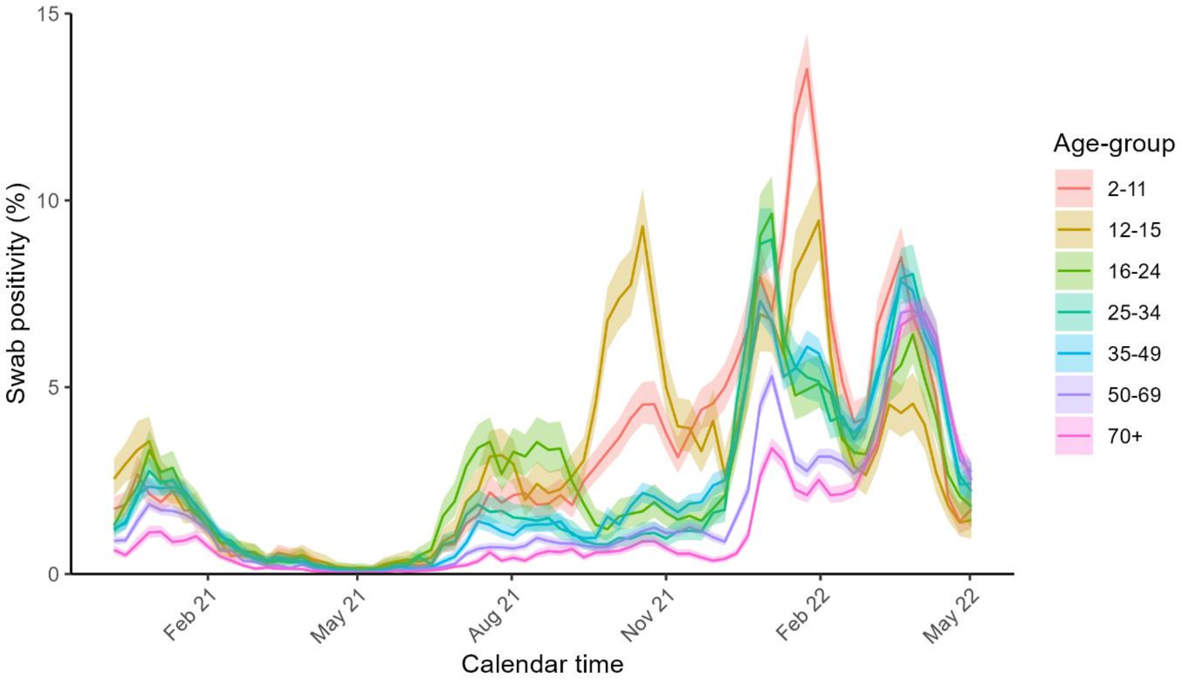

Fig. S2. Post-stratified estimate of swab positivity by age-group over time. Estimates are post-stratified for age, sex, CIS area, ethnicity, and vaccination status. Estimates are presented as posterior medians (solid lines) with shading representing 95% credible intervals.

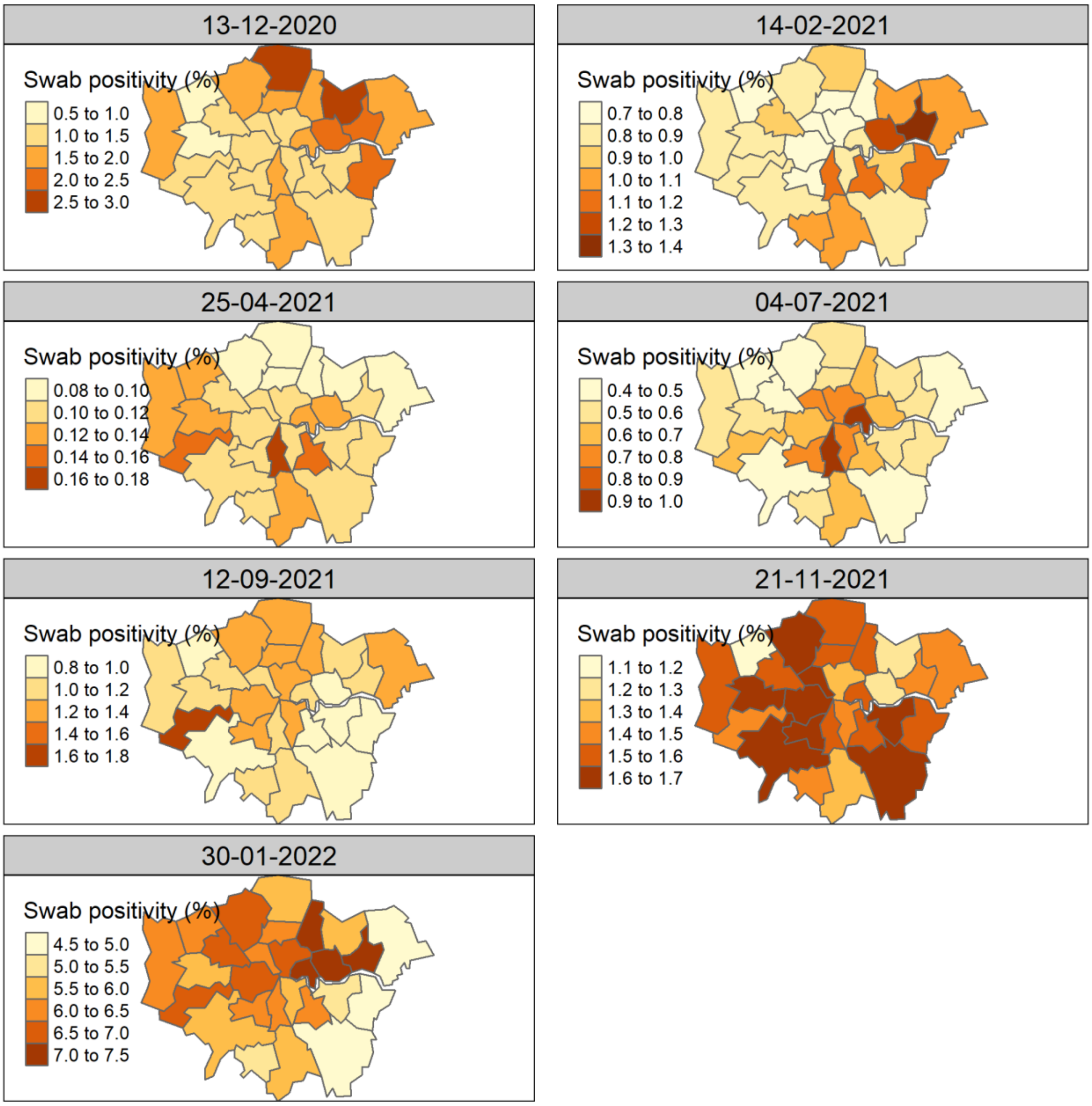

Fig. S3: Post-stratified estimate of swab PCR positivity by CIS area in London over time. Estimates are post-stratified for age, sex, CIS area, ethnicity and vaccination status.

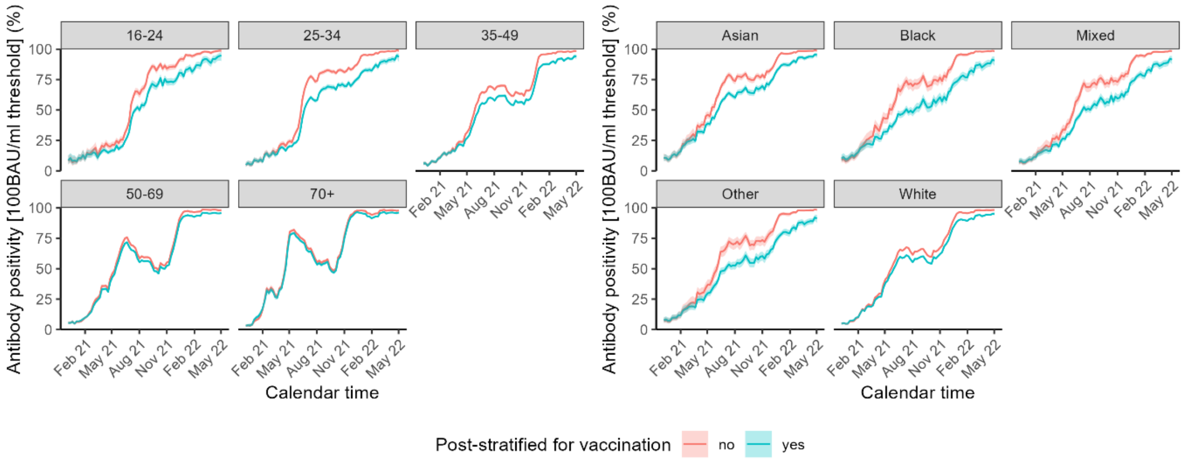

Fig S4. Impact of post-stratifying for vaccination status (yes/no and interaction with time) on estimated antibody positivity at the 100 BAU/ml threshold by age and ethnicity over time. Estimates are post-stratified for age, sex, CIS. Estimates are presented as posterior medians (solid lines) with shading representing 95% credible intervals.
