# Supplementary material for: Improving the representativeness of UK’s national COVID-19 Infection Survey through spatio-temporal regression and post-stratification
Source: Nat Commun. 2024 Jun 24;15:5340. doi: 10.1038/s41467-024-49201-4 (PMC11196632; doi:10.1038/s41467-024-49201-4)
Supplement: Supplementary file 3 — Description of Additional Supplementary Files [file 41467_2024_49201_MOESM3_ESM.pdf]

Description of Additional Supplementary Files

Supplementary Data 1. Post-stratified estimates of antibody and swab (PCR) positivity in England overall and by region (9 regions), CIS area (116 areas), age-group, and ethnicity

Estimates are post-stratified for age, sex, CIS area, ethnicity and vaccination status. Estimates for antibody levels are for those aged 16years and older; estimates for swab (PCR) positivity are for those aged 2years and older. When using these estimates please cite the paper: Pouwels et al. Improving the representativeness of UK's national COVID-19 Infection Survey through spatio-temporal regression and post-stratification.
